# Self-Assembly 2D Ti_3_C_2_/g-C_3_N_4_ MXene Heterojunction for Highly Efficient Photocatalytic Degradation of Tetracycline in Visible Wavelength Range

**DOI:** 10.3390/nano12224015

**Published:** 2022-11-15

**Authors:** Chunmin Li, Changjie Kan, Xiangtai Meng, Mengxue Liu, Qianqian Shang, Yikai Yang, Yu Wang, Xiaoxue Cui

**Affiliations:** College of Chemistry and Chemical Engineering, Liaocheng University, Liaocheng 252000, China

**Keywords:** Ti_3_C_2_ MXene, g-C_3_N_4_, 2D catalysis, tetracycline degradation

## Abstract

An ultrathin 2D Ti_3_C_2_/g-C_3_N_4_ MXene (2D-TC/CN) heterojunction was synthesized, using a facile self-assembly method; the perfect microscopic-morphology and the lattice structure presented in the sample with a 2 wt% content of Ti_3_C_2_ were observed by the field-emission scanning electron microscopy (SEM) and transmission electron microscope (TEM). The optimized sample (2-TC/CN) exhibited excellent performance in degrading the tetracycline (TC), and the degradation rate reached 93.93% in the conditions of 20 mg/L, 50 mL of tetracycline within 60 min. Except for the increased specific-surface area, investigated by UV-vis diffuse reflectance spectra (UV-vis DRS) and X-ray photoelectron microscopy (XPS) valence spectra, the significantly enhanced photocatalytic activity of the 2-TC/CN could also be ascribed to the formation of Ti-N bonds between Ti_3_C_2_ and g-C_3_N_4_ nanosheets, which reduced the width of the band gap through adjusting the position of the valence band, thus resulting in the broadened light-absorption. Furthermore, the facilitated electron transmission was also proved by time-resolved photoluminescence (TRPL) and electrochemical impedance spectroscopy (EIS), which is effective in improving the quantum efficiency of photo-generated electrons. In addition, the resulting radical-capture experiment suggested that superoxide radicals have the greatest influence on photodegradation performance, with the photodegradation rate of TC reducing from 93.16% to 32.08% after the capture of superoxide radicals, which can be attributed to the production of superoxide radicals only, by the 2-TC/CN composites with a high conduction-band value (−0.62 eV). These facilely designed 2D Ti_3_C_2_/g-C_3_N_4_ composites possess great application potential for the photodegradation of tetracycline and other antibiotics.

## 1. Introduction

With the rapid development of human society, the increasing abuse of antibiotics is becoming serious. Those antibiotics which originate from large-scale farms, hospitals, pharmaceutical factories, and domestic sewage have caused tremendous pollution in natural waterbodies [1]. Tetracycline, a broad-spectrum antibiotic, has become a common detection indicator in water. Even at low levels (<1.0 ppm), long-term exposure to residual tetracycline may induce antibiotic resistance and harm human health [2,3]. In addition, the residual tetracycline will not only affect the growth and metabolism of microorganisms, but also the microbial-community structure of the ecosystem [4]. Therefore, it is of great urgency to develop an efficient and economical strategy to degrade residual tetracycline in water.

Up until now, abiotic and biological processes, as well as combined processes have been widely reported in the degradation of tetracycline. The abiotic processes include hydrolysis [5], adsorption [6], photocatalytic oxidation [7], electro-chemistry [8], ozonation [9], and hydrogen peroxide oxidation [10]. Pure culture fungi [11] and sludges [12], sedimentation [13], and bacteria [14] are connected to the biodegradation of tetracycline. Meanwhile, the combined process has made use of photocatalysis and biodegradation [15], ozone and activated sludge [16]. However, the traditional biodegradation methods cannot effectively eliminate tetracycline, due to the antibacterial nature of antibiotics [17]. On the other hand, photocatalysis has proved to be a suitable alternative for the efficient degradation of tetracycline in water, due to its green, low cost, highly sustainable, simple reaction conditions, without causing secondary pollution [18,19,20].

The two-dimensional (2D) materials were firstly discovered in 2004, and attracted a great deal of research, based on their extraordinary photoelectronic and electrochemical properties [21]. The extraordinary electrical conductivity can be attributed to the ultrathin 2D region, which endows them with enormous potential in photocatalysis for enhancing photogenerated charge-carrier-separation efficiency [22]. Moreover, the high specific surface-area of 2D materials usually serves as a substrate for catalysts, and prevents the aggregation of catalysts [22]. Lately, metal-free g-C_3_N_4_ has been selected for the degradation of tetracycline, based on its excellent structural stability and high photocatalytic capacity [23,24]. According to reference [25], the synthesis of g-C_3_N_4_ through the thermal decomposition of urea, melamine, and thiourea methods is simple and low-cost. The semiconductor band-gap of g-C_3_N_4_ is 2.7 eV, corresponding to an optical wavelength of 460 nm, and the conduction band (CB) is −1.3 eV [26], which results in a small thermodynamic driving-force for water or organic-pollutants oxidation. Based on the excellent photoelectric performance, g-C_3_N_4_ has been widely used; for example, photocatalytic water-splitting for H_2_ and O_2_ generation, photocatalytic reduction of CO_2_ for renewable hydrocarbon fuels, photocatalytic degradation of pollutants and for bacteria disinfection, gas-phase degradation of pollutants, liquid-phase degradation of pollutants, bacterial disinfection, sensing, and so on [27,28,29]. In addition, the g-C_3_N_4_ with 2D morphology, which was caused by the weak van der Waals force between adjacent layers, provided abundant active sites for contact with the target contaminants. However, the photocatalytic efficiency of g-C_3_N_4_ is significantly declined by low rapid-charge-recombination [30], surface area [24] and limited visible-light absorption [31], etc. Methods such as heterojunction [31], co-catalyst introduction [32], element doping [33], and morphology control [34], etc., were explored, to overcome those shortcomings. For example, Xu et al. [20] synthesized the g-C_3_N_4_/MXene/Ag_3_PO_4_ heterostructure using the self-assembly route to enhance the photocatalytic degradation of tetracycline. In reference [20], Xu et al. considered only the traditional methods of modifying g-C_3_N_4,_ rather than preparing raw materials into specific shapes (as 2D or a 2D nanosheet); in addition, the raw material Ag_3_PO_4,_ containing the noble-metal element functions only as a medium for electronic transport, which partly causes a waste of resources.

In recent years, a novel 2D-transition metal carbide or nitride, which is called MXene, has been widely studied in the photocatalysis field. It is obtained by chemical etching from the MAX phase, and the general formula can be expressed by M_n+1_X_n_T_x_ [35], where M represents the early transition metal (such as Ti, V, Zr and Nb), X stands for C and/or N elements, and T symbolizes the surface functional-groups, (e.g., -O, -OH and -F). These surface-rich functional groups not only provide great convenience for compounding with other functional materials, but also make the layer spacing and composition easier to adjust. The theoretical calculation conclusion obtained from Rina Ibragimova et al. [36] evidenced that the pH and temperature have limited effects on the tunability of the surface composition, and do not lead to dramatic changes in the electronic properties of MXene, which further shows the reliability of the surface modification of MXene. As an emerging 2D material, MXene showed prominent cocatalyst effects for photocatalysis, attributed to its excellent metal conductivity, hydrophilic functionalities, fabulous reactivity and hydrophilicity [37]. Since 2014, MXene-based photocatalytic catalysts have been widely applied in N_2_ fixation [38], pollutant degradation [39], CO_2_ reduction [40], and water splitting [41]. These extensive applications evidence that MXene could enhance photocatalytic activity by playing different roles, such as promoting photogenerated charge-carrier-separation, acting as robust support, limiting photocatalyst size, and enhancing reactant adsorption.

Therefore, based on the synergistic effect of the 2D materials and the heterostructure, the new composite named 2D Ti_3_C_2_/g-C_3_N_4_ MXene composite was synthesized by introducing 2D-Ti_3_C_2_ MXene onto the surface of 2D g-C_3_N_4,_ via a facile liquid self-assembly method. The introduction of 2D-Ti_3_C_2_ MXene not only improves the electronic-transmission performance of 2D g-C_3_N_4_, but also broadens the wavelength range of visible-light absorption waves in the photocatalytic-degradation process of tetracycline. Moreover, 2D-Ti_3_C_2_ MXene also reduces the recombination of photogenerated electrons of 2D g-C_3_N_4_ and improves its recyclability and degradation efficiency during the removal of tetracycline from wastewater. Experimental results evidence that the 2D Ti_3_C_2_/g-C_3_N_4_ MXene composite shows excellent application prospects for the photodegradation of tetracycline.

## 2. Experimental Section

### 2.1. Materials

Melamine, silver nitrate (AgNO_3_), EDTA disodium saltdihydrate (EDTA-2Na), sodium sulfate (Na_2_SO_4_), benzoquinone (PBQ), tetracycline (TC), Lithium fluoride (LiF) and chloroplatinic acid (H_2_PtCl_6_) were supplied by Shanghai Macklin Biochemical Co., Ltd. (Shanghai, China). Tertbutanol (TBA), ammonium chloride (NH_4_Cl), ethanol (EtOH) and methanol (MeOH) were purchased from Aladdin-Holdings Group. 5,5-Dimethyl-1-pyrroline N-oxide (DMPO) was purchased from Beiren chemical technology Co., Ltd. (Beijing, China). Ti_3_AlC_2_ MAX was obtained from Foshan Xinxi technology Co., Ltd. (Foshan, China). Hydrochloric acid (HCl) was purchased from Yantai Yuandong fine chemical Co., Ltd. (Yantai, China). Ultrapure water was used throughout the experiment. All chemicals in this work were used without additional purification.

### 2.2. Preparation of Protonated 2D-C_3_N_4_ Nanosheet

The typical synthesis method of protonated g-C_3_N_4_ involves the calcination of melamine, following the above steps. Step 1: 3 g melamine powder was put into a crucible with a cover, and then calcinated at 550 °C for 4 h with a heating rate of 5 °C/min in a muffle furnace, under an air atmosphere. Step 2: after being naturally cooled down, the yellow bulk C_3_N_4_ powder was obtained after careful grinding. Step 3: the g-C_3_N_4_ nanosheets were dispersed into a 100 mL 0.5 M HCl, and ultrasound was performed for 0.5 h. Step 4: the obtained g-C_3_N_4_ HCl solution was washed with deionized water several times until the solution pH was 4, and the protonated g-C_3_N_4_ was obtained.

### 2.3. Preparation of 2D-Ti_3_C_2_ MXene

Step 1: 1.6 g of LiF was added into 20 mL 12 M of HCl, and stirring started at 500 r/min for 10 min, to completely dissolve LiF, finally forming an etching solution. Then, 1.0 g Ti_3_A1C_2_ was gradually added to the etching reagent, and the reaction temperature was set at 45 °C, with a reaction time of 45 min. Step 2: after the etching, the black liquid was centrifuged at 3000 r/min. The original etching reagent was poured out, and the bottom precipitation was collected. The collected precipitation was washed again with 1M HCl several times to absorb the excess LiF and other impurities, until the superstratum was colorless and transparent. Step 3: the precipitation was washed with deionized water for 3–5 times. The upper layer of the liquid gradually became cloudy and stratified, at which point the MXene began to stratify. Due to the low concentration of this part of the solution, further centrifugation was required, to obtain the bottom precipitated portion. Finally, water continued to be added, and it was centrifuged until the pH reached approximately 6. Step 4: the lower layer of precipitation was ultrasonically treated for 30 min, during which inert protective gas was added and ice was added to the ultrasonic groove, to avoid irreversible damage to the structure of the material during ultrasonic heating. After the ultrasound, the material was centrifuged for 40 min at a speed of 10,000 r/min, and the precipitation was freeze-dried for 48 h at −50 °C, to obtain powder 2D-Ti_3_C_2_ MXene.

### 2.4. Synthesis of 2D Ti_3_C_2_/g-C_3_N_4_ MXene Composite

**Scheme 1:**
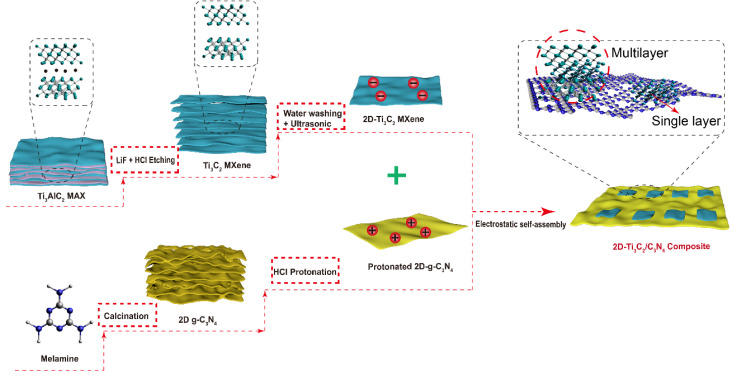
Schematic diagram of the preparation process of 2D Ti_3_C_2_/g-C_3_N_4_ MXene composite materials.

Step 1: the protonated 2D-C_3_N_4_ was dispersed into 100 mL deionized water and stirred magnetically for 10 min. Then, 1 mL 2 g/L of single-layer Ti_3_C_2_ MXene solution was added into 100 mL protonated 2D-C_3_N_4_ suspension, drop by drop, and stirred for 30 min after dropping; the suspension was then static for 1 h. Step 2: The suspension containing Ti_3_C_2_/C_3_N_4_ composite was centrifuged, and the Ti_3_C_2_/C_3_N_4_ precipitation was obtained by pouring out the supernatant. Step 3: the Ti_3_C_2_/C_3_N_4_ precipitation was placed in a vacuum drying oven and dried at 80 °C for 24 h, to obtain a 2D Ti_3_C_2_/g-C_3_N_4_ composite with a Ti_3_C_2_ content of 2 wt%, which can be represented as 2-TC/CN. The TC/CN composites with Ti_3_C_2_ content of 1 wt%, 3 wt% and 5 wt% were prepared using the same method, which was expressed as 1-TC/CN, 3-TC/CN and 5-TC/CN, respectively. The synthesis process is illustrated in Scheme 1.

### 2.5. Characterization

The microscopic morphology of the prepared samples was analyzed using field-emission scanning electron microscopy (FESEM) (Hitachi, S-4800, Tokyo, Japan). The lattice structure and energy-dispersive X-ray spectroscopy (EDS) images were conducted on a transmission electron microscope (TEM), (Philips, JEOL JEM-2010F, Nieuw-Vennep, The Netherlands), with an accelerating voltage of 100 kV. The crystal structure of the as-synthesized samples was studied by X-ray diffraction (XRD) analysis (Rigaku, SMARTLAB3KW, Tokyo, Japan) on the PERSEE XD3 diffractometer system from Bruker with Cu-Kα radiation (λ = 1.5420 Å), scanning-angle range purview 5–80° at 45 kV and 200 mA. The analysis of the functional group of as-prepared samples was conducted on the infrared diffuse reflectance (FT-IR) spectrum (Thermo Fisher Scientific, IS50, Waltham, MA, USA) with an in situ cell. The chemical state and valence-band spectra of the prepared samples were characterized using X-ray photoelectron microscopy (XPS) (Thermo Fisher Scientifc, ESCALAB 250Xi, Waltham, MA USA) spectra, using a monochromated Al X-ray resource, at 1486.6 eV. The electrical properties and the and capture of superoxide radicals and hydroxyl radicals were measured by the electron spin resonance (ESR) spectra (JEOL, JES-FA200, Tokyo, Japan). Photoluminescence (PL) spectral measurements for analyzing the photogenerated electron-hole complexation rate of the materials were performed on a Fluorolog-3 fluorescence spectrophotometer with excitation wavelength of 464 nm (Hitachi, F-2700, Tokyo, Japan). The values of the energy gap of the as-synthesized samples were evaluated by UV-vis diffuse reflectance spectra (UV-vis DRS) using a UV-vis spectrophotometer with excitation wavelength of 365 nm (Shimadzu, UV-3600, Kyoto, Japan). The separation efficiency and transient photocurrent of the samples were tested by time-resolved photoluminescence (TRPL) spectra on a fluorescence lifetime spectrophotometer (FLS1000, Edinburgh, UK). The enhancement of conductivity of the composites was measured by electrochemical impedance spectroscopy (EIS) on an electrochemical workstation (CHI660E, Shanghai Chenhua, China).

### 2.6. Working Electrode Preparation Procedure

The working electrode was prepared by a dip-coating method. Specifically, 10 mg of the sample and 500 μL of 5 wt% Nafion solution were dispersed in 5 mL of a mixed solution of water and ethanol (V_water_/V_ethanol_ = 1/4), to form a homogeneous slurry, which was subsequently dropped and coated onto a 1.0 cm × 1.0 cm ITO glass substrate and then exposed to air for 6 h, to dry.

### 2.7. Photoelectrochemical Measurement

Photoelectrochemical measurements were carried out on an electrochemical workstation in 0.5 M Na_2_SO_4_ aqueous solution with the standard three-electrode system. The saturated calomel electrode (SCE) and platinum wire served as the reference electrode and counter electrode, respectively. The working electrode was prepared by the doctorblade technique on FTO conductive glass (MTI Corporation, Baiye Center, China), which was described in our previous work. The visible light was provided by a 300 W xenon lamp (Zhongjiao Jinyuan Technology Co., Ltd., CEL-HX300, Beijing, China) equipped with a cutoff filter (>420 nm). Prior to measurement, the suspension was purged with N_2_ several times, to remove the residual air.

### 2.8. Evaluation of Photocatalytic Degradation of Tetracycline

The photocatalytic activity of the samples was evaluated by the decomposition of the liquid tetracycline (TC, 20 mg/L, 50 mL) under a 300 W xenon lamp equipped with a cutoff filter (>420 nm). Prior to irradiation, the mixed suspension was stirred in the dark for 30 min, to achieve the adsorption-desorption equilibrium. At given time-intervals, 3 mL liquor was extracted, then high-speed centrifuged (17,000 r/min) for 10 min, to remove the catalyst completely. The concentration of TC was analyzed using a UV-Vis spectrophotometer (Shimadzu, UV-Vis 2700, Kyoto, Japan).

Free radical trapping tests: to further investigate the photocatalytic oxidation pathway, 2-TC/CN sample was taken as a photodegradation catalyst. Tert-Butyl alcohol (TBA, 10 mM, 5 mL) as •OH radical scavenger, EDTA-2Na (0.1 g) as hole scavenger, and p-Quinone as •O^2−^ scavenger were chosen, respectively, to participate in the RhB photodegradation test. The process was the same as that of the photocatalytic degradation of the TC above, except for the extra added corresponding scavengers.

## 3. Results and Discussion

### 3.1. Synthesis Process of 2D Ti_3_C_2_/g-C_3_N_4_ MXene Composite

The formation process of the 2D Ti_3_C_2_/g-C_3_N_4_ MXene composite was described in Scheme 1. The 2D-Ti_3_C_2_ was etched from the starting sample Ti_3_AlC_2_ MAX by the following equations:

(1) LiF+HCl→LiCl+HF

(2) $${\mathrm{Ti}}_{3}{\mathrm{AlC}}_{2\;}+3\mathrm{HF}\to {\mathrm{Ti}}_{3}C_{2}\left(\mathrm{multilayer}\right){+\mathrm{AlF}}_{3}+\frac{3}{2}H_{2}\;$$

(3) $${\mathrm{Ti}}_{3}C_{2}\left(\mathrm{multilayer}\right){+3\mathrm{Li}}^{+}\left(\mathrm{intercalator}\right)\to {\mathrm{Ti}}_{3}{\mathrm{Li}}_{3}C_{2}\left(\mathrm{monolayer}\right)$$

(4) $${\mathrm{Ti}}_{3}{\mathrm{Li}}_{3}C_{2}{+H}_{2}O\to {\mathrm{Ti}}_{3}C_{2}{(\mathrm{monolayer})+3\mathrm{Li}}^{+}$$

**Figure 1:**
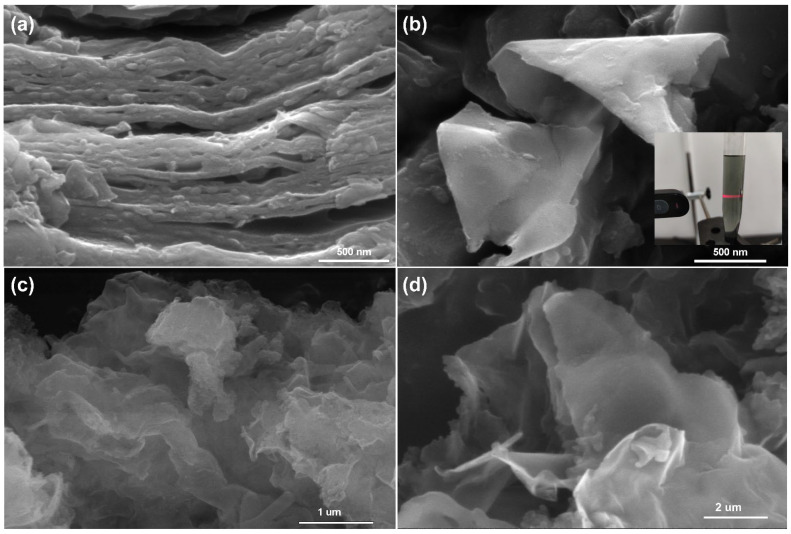
FESEM images of (**a**): multilayer Ti_3_C_2_ MXene, (**b**): monolayer Ti_3_C_2_ MXene and the Tyndall effect in illustration, (**c**): sheet g-C_3_N_4_, and (**d**): 2-TC/CN.

Based on the SEM images, we can see that the Ti_3_AlC_2_ MAX with bulk morphology (Figure S1) changed into accordion fold (Figure 1a) after being etched by the concentrated hydrochloric acid solution of lithium fluoride, which proved the efficient removal of aluminum ions from the MAX phase. The morphology and EDS-mapping of the composite (2-TC/CN) is shown in Figure S2. As shown in Figure 1b, the accordion fold-like Ti_3_C_2_ MXene was changed into monolayer Ti_3_C_2_ MXene after the dispersion of the aqueous solution and ultrasonic vibration, which can be evidenced by the Tyndall phenomenon exhibited in the illustration in Figure 1b. As shown in Figure 1c, the morphology of the g-C_3_N_4_ maintained a sheet structure after ultrasonic treatment in aqueous HCl solution. The morphology of the 2D-TC/CN composites is exhibited in Figure 1d, where we can see that the morphology of protonated g-C_3_N_4_ with a 2% content of monolayer Ti_3_C_2_ MXene was not distinguished from that of the protonated g-C_3_N_4_. This could be attributed to the thin-flake morphology of both the 2D-TC/CN composites and the protonated g-C_3_N_4_, and it is hard to distinguish from the SEM images. The SEM images of Figure 1d show that the 2-TC/CN composites exhibited a more perfect two-dimensional layered morphology than composites with other contents of the monolayer Ti_3_C_2_ (Figure S3).

**Figure 2:**
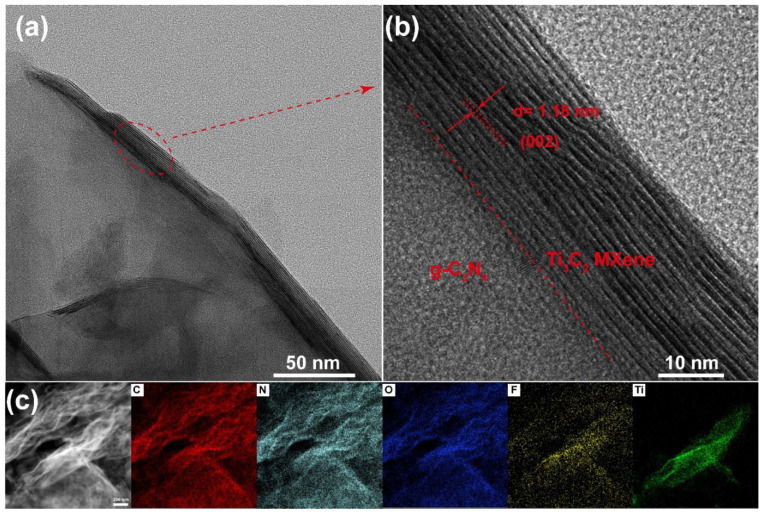
(**a**) TEM image of 2-TC/CN, (**b**) HRTEM image of 2-TC/CN, (**c**) EDS mapping images of C, N, O, F, and Ti elements.

To throw light on the morphology and microstructure of the 2D-TC/CN composites, the 2-TC/CN sample was chosen to be further investigated, using TEM and HRTEM (Figure 2). The SEM image of the 2D-TC/CN in Figure 1a shows that the 2D-TC/CN composites still maintained two-dimensional structures consisting of flat, regularly shaped nanosheets of 2D/2D structures. Figure 2b exhibits clear lattice-fringes and a disordered area, between which an intimate boundary arises. The disordered area was assigned to the g-C_3_N_4_ nanosheets, while the clear lattice-fringe was the Ti_3_C_2_ flakes, and the obvious lattice-spacing of 1.18 nm was attributed to the (0 0 2) facet of Ti_3_C_2_ [42]. The intimate boundary suggested the successful synthesis of the 2D-TC/CN composite, which is in accordance with the EDS analysis in Figure 2c. Figure 2c evidences the fact that the elements C, N, O, and F were uniformly distributed on the composites, and that the Ti element only existed in the 2D-Ti_3_C_2_ section, creating a clear boundary between the distribution of other elements, which corresponded to the results in Figure 2b. This intimate boundary was beneficial to the transfer of photoinduced electrons from g-C_3_N_4_ to Ti_3_C_2,_ due to the excellent electronic conductivity of Ti_3_C_2_.

### 3.2. Structure Characterization of 2D Ti_3_C_2_/C_3_N_4_ MXene Composite

**Figure 3:**
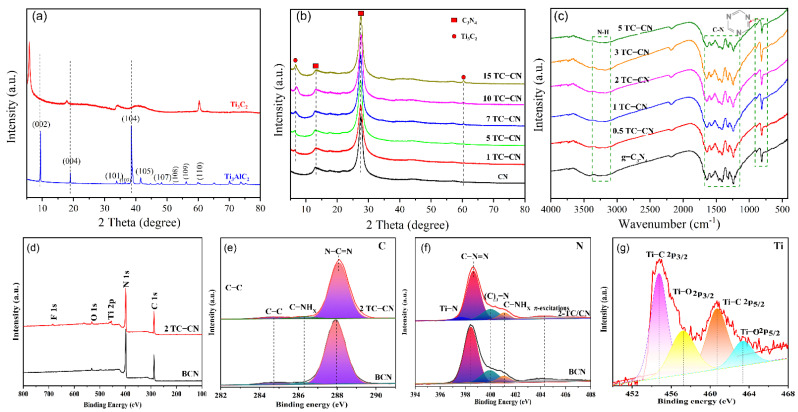
(**a**) XRD patterns of Ti_3_AlC_2_ and Ti_3_C_2_, (**b**) XRD patterns of g-C_3_N_4_ and TC/CN, (**c**) FTIR spectra of g-C_3_N_4_ and TC/CN, (**d**) XPS survey of g-C_3_N_4_ and 2-TC/CN, (**e**) high-resolution XPS spectra of C1s, (**f**) high-resolution XPS of N1s, (**g**) high-resolution XPS of Ti 2p.

The phase structure of the as-synthesized samples was characterized by the X-ray diffraction method. As shown in Figure 3a, the XRD pattern with intense peaks at 9.6° and 39°, which respectively correspond to the (0 0 2) and (1 0 4) facets, is attributed to the Ti_3_AlC_2_ MAX phase [43]. After etching treatment with the concentrated hydrochloric acid solution of lithium fluoride, the (0 0 2) facet at 9.6° and (0 0 4) facet at 19.3° of Ti_3_AlC_2_ shifted to lower angles in the XRD pattern of Ti_3_C_2_. Moreover, the most intense peak at 39° disappeared after etching treatment. According to previous studies [44,45], those changes that appeared in Figure 3a suggest the successful conversion from Ti_3_AlC_2_ to Ti_3_C_2_. The XRD patterns of g-C_3_N_4_ and 2D-TC/CN composites in Figure 3a,b show that the two distinct diffraction peaks at 13.1° and 27.5° corresponded to the (1 0 0) and (0 0 2) facets of g-C_3_N_4_, respectively, and corresponding to the intralayer packing of the polymeric melon units and the interlayer π-π stacking of g-C_3_N_4_, respectively [46]. When the content of Ti_3_C_2_ > 10 wt%, two new diffraction peaks at 6.6° and 60.4° gradually appeared in the TC/CN composites, which corresponded to (the 0 0 2) and (1 1 0) facets of Ti_3_C_2_, respectively. No changes occurred on the site of the two peaks of g-C_3_N_4_, even after loading Ti_3_C_2,_ suggesting the combination of g-C_3_N_4_ and Ti_3_C_2_ belonged to an interfacial self-assembly between monolayers.

The FTIR results of the prepared samples are exhibited in Figure 3c. The broad peaks between 3000 and 3500 cm^−1^ represent N-H stretching vibrations [40], and the intensity of the peak was slightly reduced after loading Ti_3_C_2_ in the protonated g-C_3_N_4,_ which suggests that the chemical N-H bond might act as “positive bridge” to attract electronegative Ti_3_C_2_ through electrostatic self-assembly. The several peaks at 1200–1700 cm^−1^ suggest the typical stretching modes of CN heterocycles, such as C-N and C=N heterocycles [47]. The peak at approximately 810 cm^−1^ belonged to the out-of-plane breathing vibration of the triazine units [48]. From Figure 3c, we can see that the 2D-TC/CN composites with different contents of Ti_3_C_2_ almost process the same FTIR spectra, indicating that the composites maintain a stable structure during the compounding process.

The chemical states and compositions of g-C_3_N_4_ and 2-TC/CN were explored by XPS. As shown in Figure 3d, the elements of C 1s, N 1s, O 1s, F 1s and Ti 2p, which are clearly presented in g-C_3_N_4_ and Ti_3_C_2_, suggest the successful synthesis of the 2D-Ti_3_C_2_/C_3_N_4_ composites. The high resolutions of C 1s, N 1s and Ti 2p between g-C_3_N_4_ and Ti_3_C_2_ are shown in Figure 3e–g. For C 1s high resolutions, the binding energy at 284.7 eV was attributed to C-C on the g-C_3_N_4_ and 2-TC/CN surface [49], the peaks at 286.3 eV (2-TC/CN) and 286.2 eV (g-C_3_N_4_) were attributed to C-NH_X_ [50] and the peaks at 288.1 eV (2-TC/CN) and 287.9 eV (g-C_3_N_4_) were assigned to C-N=C [51]. For N 1s in Figure 3f, the high resolutions of N 1s in 2-TC/CN can be divided into five peaks, while N 1s in g-C_3_N_4_ can be divided into four peaks. The peaks at 398.6 eV (2-TC/CN) and 398.4 eV (g-C_3_N_4_) were attributed to N-C=N (triazine rings), the peak at 400 eV (both in 2-TC/CN and g-C_3_N_4_) was assigned to (C)_3_-N, the peaks at 401.1 eV (2-TC/CN) and 401 eV (g-C_3_N_4_) were attributed to C-NH_X_ [52,53], the peak at 404.3 eV (both in 2-TC/CN and g-C_3_N_4_) was attributed to charging effects or positive-charge location in heterocycles [50]. Notably, the newly generated peak at 397.6 eV presented in 2-TC/CN was assigned to Ti-N interaction [54]. For Ti 2p in Figure 3g, the Ti 2p spectrum was deconvoluted into four components, including peaks at 454.8 eV, 457.2 eV, 460.7 eV and 463.4 eV. The peaks at 454.8 eV and 460.7 eV were assigned to Ti-C 2p_3/2_ and Ti-C 2p_5/2_ [55], while peaks at 457.2 eV and 463.4 eV were attributed to Ti-O 2p_3/2_ and Ti-O 2p_5/2_ [56]. From the high-resolution spectra of g-C_3_N_4_ and 2-TC/CN in Figure 3e–g, we can see that the binding energy of C-NHX, C-N=C and C-N=C in the 2-TC/CN composite exhibited a positive shift, indicating the low electrondensity of g-C_3_N_4_ in 2-TC/CN and the strong interaction between g-C_3_N_4_ and Ti_3_C_2_. Through the electron density of the C-NH_X_ and the newly generated Ti-N bond in the 2-TC/CN, it can be deduced that Ti_3_C_2_ showed self-assembly formation on the g-C_3_N_4_ through Ti-NH_X_ interaction, as shown in Scheme 1.

### 3.3. Optical and Electrochemical Properties of 2D Ti_3_C_2_/g-C_3_N_4_ MXene Composite

**Figure 4:**
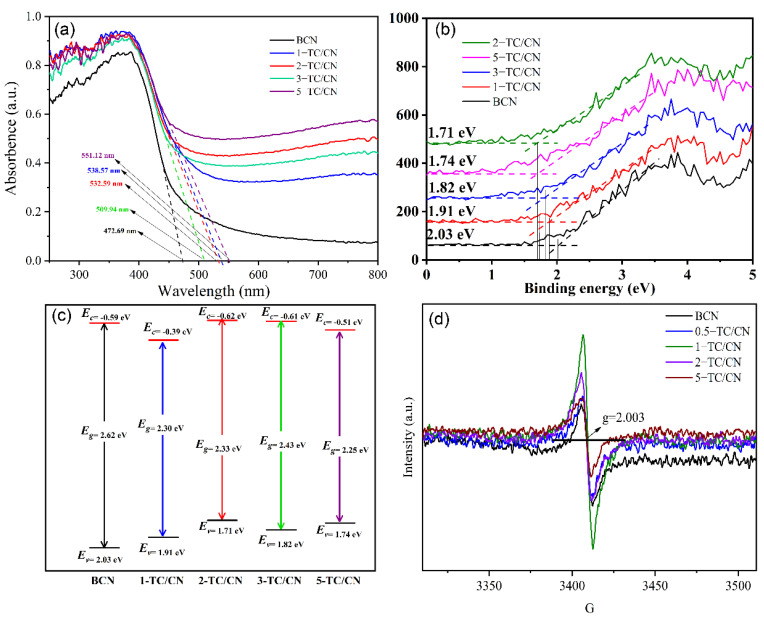
Diffuse reflectance spectra of g-C_3_N_4_ (**a**), XPS valence-band spectra (**b**), band structure (**c**) and electron paramagnetic resonance (EPR) spectra (**d**), of g-C_3_N_4_ and (1-5)-TC/CN.

The light-absorption capacity of the as-prepared samples was evaluated by the UV-vis diffuse reflectance spectra. It can be seen from Figure 4a that the absorption edge of the g-C_3_N_4_ sample was 472.69 nm. The optical-absorption properties of the TC/CN composites gradually improved optical absorption in the range of visible light (510–551 nm), accompanied by the increased content of Ti_3_C_2_ from 1 wt% to 5 wt%. This change may be attributed to the dark slate-gray color of Ti_3_C_2,_ which possessed full-spectrum absorption. The improved optical absorption can be conducive to converting the light quantum into heat energy and enhancing the photocatalytic reaction, due to the efficient photothermal-conversion characteristics [57]. The valence-band values (Ev) of g-C_3_N_4_ and 1-TC/CN, 2-TC/CN, 3-TC/CN, and 5-TC/CN were measured using XPS valence-band spectra. As described in Figure 4b, the valence-band values of g-C_3_N_4_ and 1-TC/CN, 2-TC/CN, 3-TC/CN, and 5-TC/CN were 2.03 eV, 1.91 eV, 1.71 eV, 1.82 eV and 1.74 eV, respectively. Based on the valence-band values, the lowest value (1.71 eV) represented the optimal valence-band value appearing in the 2-TC/CN sample. Moreover, based on the results in the diffuse-reflectance spectra, the band-gap (Eg) value of g-C_3_N_4_ was 2.62 eV, which could be calculated using the Tauc plot method (Equation (5)). Using the same method, the band-gap (Eg) values of 1-TC/CN, 2-TC/CN, 3-TC/CN, 5-TC/CN were 2.30 eV, 2.33 eV, 2.43 eV and 2.23 eV, respectively, and the computation procedure is listed in Table S1. Based on the values of the band gap, we can conclude that the band gap of the composites reduced when compared to that of g-C_3_N_4_, resulting in the excellent absorption edge of the light. Thus, according to the results in Figure 4a, the introduction of Ti_3_C_2_ into g-C_3_N_4_ not only enhanced the light-absorption capacity, but also improved the oxidant capacity of the composites, and these two changes synergistically improved the photocatalytic performance of the materials. The conduction-band (CB) values of g-C_3_N_4_ and 1-TC/CN, 2-TC/CN, 3-TC/CN, and 5-TC/CN can be calculated using Equation (6) and the results are presented in Figure 4c. The expression of Equations (5) and (6) are as follows:

(5) Eg(eV)=1240γ

(6) $$E_{c}(\mathrm{eV})=\;E_{v}-E_{g}$$

where *γ* (nm) represents the absorption edge of the samples, *E_g_* (eV) is the value of the band gap, and *E_c_* (eV) is the value of the conduction band.

The introduction of Ti_3_C_2_ into g-C_3_N_4_ greatly impacted the electrical properties of the TC/CN composites, and the intrinsic reason was explored using the room-temperature EPR measurement. From Figure 4d, we can see that a single Lorentzian line is present at a g-value of 2.003 in all samples, which originates from the π-excitation of the triazine units in the g-C_3_N_4_ [58]. The intensities of (1-5)-TC/CN were higher than that of g-C_3_N_4,_ and the intensity order was 1-TC/CN > 2-TC/CN > 5-TC/CN > 0.5-TC/CN > g-C_3_N_4_. The enhanced intensities may be attributed to the new formation of the Ti-N bond, which increased the asymmetry of unpaired electrons around the π bond of the triazine units. The enhanced asymmetry of the unpaired electrons was beneficial in accelerating the electron mobility in the π bond of the triazine units in the 2D-TC/CN composites, which may be due to the delocalization of the valence electrons and the excellent electroconductibility of Ti_3_C_2_ MXene.

### 3.4. Photodegradation Performance and Mechanism of Tetracycline

**Figure 5:**
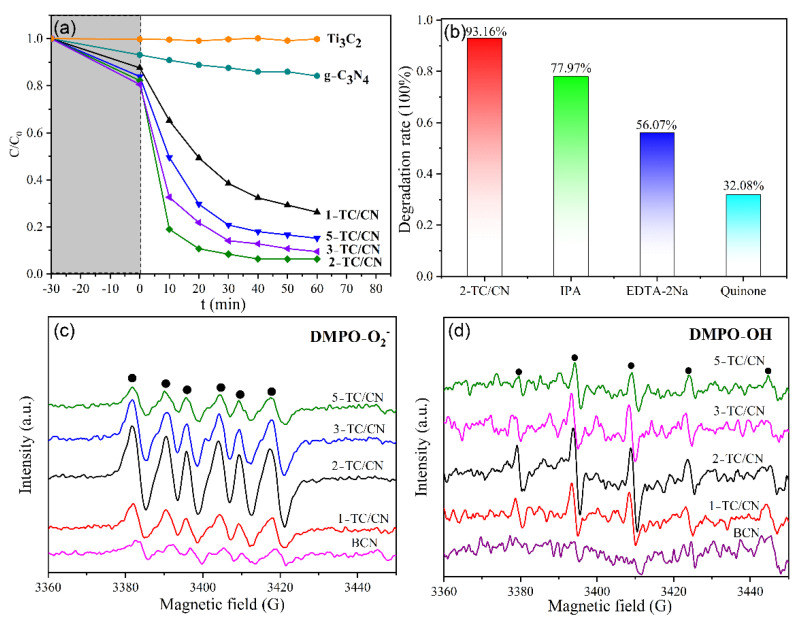
(**a**) Photodegradation of tetracycline by g-C_3_N_4_ and 1-TC/CN, 2-TC/CN, 3-TC/CN, 5-TC/CN, (**b**) the effect of IPA, EDTA-2Na and quinone on the photodegradation of tetracycline, spin-trapping EPR spectra of g-C_3_N_4_ and 1-TC/CN, 2-TC/CN, 3-TC/CN, 5-TC/CN (**c**) in aqueous dispersion and (**d**) in methanol dispersion after light irradiation, using DMPO as a radical-trapping agent.

To evaluate the photocatalytic properties of the composites, a typical tetracycline was selected as the target pollutant. In this study, the main target was to thoroughly investigate the intrinsic degradation-mechanism of tetracycline on the composites, so the degradation tests were designed at the best conditions for the target pollutant. As shown in Figure 5a, the residual rate of pure g-C_3_N_4_ was 83.92%, while the residual rate showed a marked decline with the addition of Ti_3_C_2_ into g-C_3_N_4,_ and the best photodegradation performance of tetracycline was presented in the composites with a 2 wt% content of Ti_3_C_2,_ which can be denoted as 2-TC/CN. The residual rate of tetracycline reached 6.05% within 60 min, corresponding to 93.95% of the degradation rate. These photodegradation results powerfully suggest the composites possess an excellent photodegradation performance for the degradation of tetracycline. Moreover, the reusability of the 2-TC/CN after tetracycline removal using bare adsorption and adsorption-photocatalysis synergism modes was compared, as presented in Figure S4. It is evident that the degradation rate of tetracycline shows no apparent loss after five cycles of experiments, indicating that the prepared 2-TC/CN is stable, and difficult to decompose during the process. We also made a detailed comparison of the photocatalytic activity of the degrading tetracycline of the prepared 2-TC/CN with that of the photocatalysts prepared by other groups in the literature. Through an extensive literature research, the intensify and wavelength of incident light, the distance between light source and reactor, and the addition amount of the photocatalyst are usually different, and are listed as follows (shown in Table S2). It is obvious that the prepared 2-TC/CN also exhibits the excellent photocatalytic activity of degrading tetracycline among the prepared g-C_3_N_4_-based photocatalysts.

For the purpose of gradually revealing the degradation mechanism of tetracycline by the composite materials, the basic units affecting photocatalytic performance, such as photogenerated electrons, photogenerated hole and superoxide radical, were tested in this section, and the results are listed in Figure 5b. Figure 5b suggests that the photodegradation rate of tetracycline was reduced from 93.16% to 77.97%, 93.16% to 56.07%, and 93.16% to 32.08%, respectively, after adding IPA (photogenerated-electrons scavenger), EDTA-2Na (photogenerated-hole scavenger) and quinone (superoxide-radical scavenger). Based on the results, it can be concluded that superoxide radicals have the greatest influence on photodegradation performance, followed by photogenerated holes and finally photogenerated electrons. Moreover, these results also corresponded to the fact that the introduction of Ti_3_C_2_ into g-C_3_N_4_ could enhance the transmission performance of photogenerated electrons and the electron-trapping ability, and reduce the recombination of photogenerated holes, resulting in the least impact on the photodegradation of tetrac-cline from the capture of photogenerated electrons.

The EPR technique was performed to throw light on the •O_2_^−^ and •OH radical production of 2D-TC/CN composites. Figure 5c shows the characteristic peaks of DMPO^−^•O_2_ under visible-light irradiation; the intensity of the peaks in 2-TC/CN were the highest, which implied the highest number of the generated •O_2_^−^ radical species in this sample. The rationally generated •O_2_^−^ can be attributed to the lower CB potentials (−0.62 eV, 2-TC/CN) of the 2-TC/CN when compared to the reduction potential (−0.33 eV) of O_2_/•O_2_^−^ [59]. Figure 5d exhibits the typical characteristic peaks of the DMPO^−^•O_2_ radicals in the g-C_3_N_4_ and TC/CN composites, which suggests the presence of •OH radical in the samples. Similarly to the formation of •O_2_^−^ radical, the highest number of generated •OH radical species also presented in the 2-TC/CN. However, the VB potential (1.71 eV) of the 2-TC/CN was not positive enough to form •OH radical through water (2.37 eV) or hydroxyl (1.99 eV) oxidation [60]. Thus, in line with previous studies [61,62], DMPO^−^•O_2_ peaks may be formed by the further reduction of •O_2_^−^ following the process:

•O_2_^−^→H_2_O/OH^−^→•OH

**Scheme 2:**
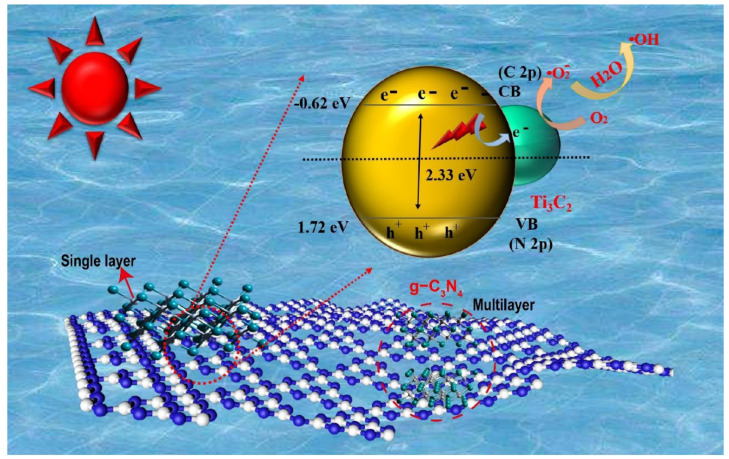
Diagram of photodegradation mechanism of tetracycline.

This process can also be verified by the above fact that the effect of photogenerated holes on degradation performance was less than that of the superoxide ions. Based on the results in Figure 4c and Figure 5c,d, the degradation process of tetracycline can be shown in the following mechanism diagram (Scheme 2):

### 3.5. Transfer Mechanism of Photogenerated Carrier

**Figure 6:**
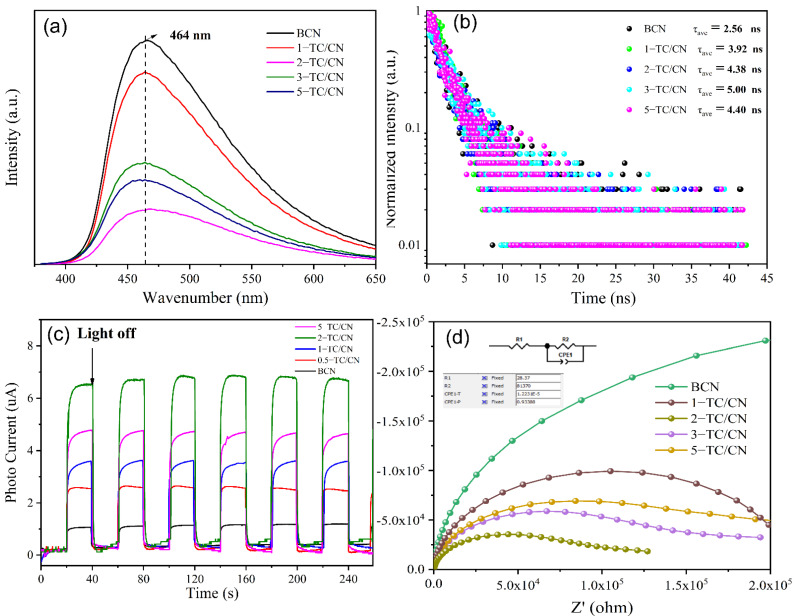
(**a**) Room-temperature PL spectra, (**b**) transient fluorescence-emission spectra, (**c**) transient photocurrent responses, (**d**) EIS spectra, of as-prepared samples.

According to the above results, the photodegradation performance of the composites mainly depends on the generated photogenerated-electrons. Thus, the transfer mechanism of the photogenerated carriers should be clarified. Steady-state photoluminescence (PL) was adopted to reveal the recombination of electrons and holes. As is known, the weaker peak-intensity in the PL spectra suggested a lower recombination of electrons and holes. Figure 6a exhibits the strongest fluorescence intensity, which appeared on the g-C_3_N_4,_ and the intensity sharply decreased with the addition of Ti_3_C_2_; this could be ascribed to the rapid electron-transfer of metallic Ti_3_C_2_ to reduce the recombination of the photogenerated carriers. The lowest PL intensity presented in the 2-TC/CN sample, which corresponded to the results in Figure 4. To further investigate the transfer of photogenerated carriers, transient fluorescence-emission spectra were used to test the lifetime of the photogenerated carriers. As illustrated in Figure 6b (the calculation procedure is listed in Table S3), the lifetime (4.38 ns, 2-TC/CN) of the photogenerated carriers of the 2D-TC/CN samples remarkably increased, compared with that (2.56 ns) of the pure g-C_3_N_4_, indicating the sluggish recombination and effective transfer of photogenerated carriers at the interface of the 2D-TC/CN samples, which was a considerable reason for the enhanced photocatalytic activity [63]. Moreover, transient photocurrent response and electrochemical impedance spectra (EIS) were applied to further confirm the excellent electron-transport properties of the composites. As shown in Figure 6c, the photocurrent of the 2-TC/CN sample was approximately six times larger than that of the pristine g-C_3_N_4_, suggesting the better charge-transfer efficiency within the ultrathin 2D-TC/CN composites. In particular, the 2-TC/CN processed the largest photocurrent, which might be due to its better crystallization, higher light-harvesting ability and more intimate heterojunction-structure between the g-C_3_N_4_ and Ti_3_C_2_, resulting in more photoinduced electrons and holes produced, and more efficient separation and immigration of photoexcited carriers. The enhanced conductivity of the 2-TC/CN composites was also verified by EIS. Figure 6d shows that the smallest Nyquist plots radius was found in the 2-TC/CN composites, indicating the best conductivity and lowest recombination-probability of the 2-TC/CN, which was in agreement with the fact that the semicircle diameter in the EIS Nyquist plot is relevant to the electron-transfer resistance [64]. The EIS plots and Rs Rct and CPE values of 2-TC/CN are well fitted by the ZView software (Version number: 3.2.0.49, Scribner Associates Inc., USA), where an equivalent circuit model is established in the illustration in Figure 6d.

## 4. Conclusions

The preparation process of the 2D Ti_3_C_2_/g-C_3_N_4_ composites suggested that self-asembly was an effective method to realize the combination of g-C_3_N_4_ and Ti_3_C_2,_ due to the electrostatic interaction between the electropositive g-C_3_N_4_ and the electronegative Ti_3_C_2_. The new Ti-N bond emerging in the 2D Ti_3_C_2_/g-C_3_N_4_ composites evidenced the fact that the composite was formed by the bonding of titanium and nitrogen. The experimental study of the photodegradation mechanism showed that the degradation of tetracycline was carried out in two ways: on the one hand, superoxide ions generated by photogenerated-electron excitation, and on the other hand, hydroxyl radicals, formed by oxidizing water molecules or hydroxyl ions through superoxide ions. The combination of superoxide and hydroxyl radicals in the degradation of tetracycline showed the practical application value of the 2D Ti_3_C_2_/g-C_3_N_4_ composites in the field of photodegradation of antibiotics. The generation and transmission of photoelectrons are the decisive factors for the efficient degradation of tetracycline. This study evidenced the fact that that the introduction of 2D-Ti_3_C_2_ MXene into g-C_3_N_4_ not only enhanced the light-absorption capacity in the visible-light range, but also improved the transmission property of the photo-induced electrons. The improvement of light-absorption performance is due to the introduction of titanium carbide, which changes the color of the composites, while the improvement of the electron-transport performance can be attributed to the excellent metal conductivity of 2D-Ti_3_C_2,_ which resulted in more photoinduced electrons and holes produced, and more efficient separation and immigration of photoexcited carriers. Moreover, the results of density-functional theory calculations further revealed the transfer of the photogenerated electrons from the 2D-C_3_N_4_ to 2D-Ti_3_C_2_, which evidenced the important synergistic effect of the 2D-Ti_3_C_2_ in improving the catalytic activity of 2D-C_3_N_4_. In short, 2D Ti_3_C_2_/g-C_3_N_4_ composites possess great application prospects for the photodegradation of tetracycline and even the antibiotics field, based on the results of the photodegradation experiment and degradation-mechanism analysis.

## Data Availability

The data presented in this study are available on request from the corresponding author.

## Supplementary Materials

The following supporting information can be downloaded at: https://www.mdpi.com/article/10.3390/nano12224015/s1, Figure S1. SEM image of Ti_3_AlC_2_ MAX phase; Figure S2. The morphology and EDS-maping of the pure sheet MXene (Ti_3_C_2_); Figure S3. SEM images of 1-TC/CN, 3-TC/CN, 5-TC/CN; Figure S4. The the reusability of 2-TC/CN MXene composite; Figure S5. UV-vis spectra of the pure Mxene (Ti_3_C_2_); Table S1. Valence band, band gap and conduction band computation procedure of the samples; Table S2. Comparison of the photocatalytic activity of degrading tetracycline of the prepared 2-TC/CN with that of photocatalysts in literature; Table S3. The calculation procedure of the lifetime of photogenerated carriers. References [65,66,67] are cited in the supplementary materials.
