# Supplementary material for: Self-Assembly 2D Ti3C2/g-C3N4 MXene Heterojunction for Highly Efficient Photocatalytic Degradation of Tetracycline in Visible Wavelength Range
Source: Nanomaterials (Basel). 2022 Nov 15;12(22):4015. doi: 10.3390/nano12224015 (PMC9699115; doi:10.3390/nano12224015)
Supplement: Supplementary file 1 [file nanomaterials-12-04015-s001.zip › nanomaterials-1981972-supplementary.pdf]

Supplementary Materials

Self−Assembly 2D Ti_3C_2/g−C_3N_4 MXene Heterojunction for Highly Efficient Photocatalytic Degradation of Tetracycline in Visible Wavelength Range

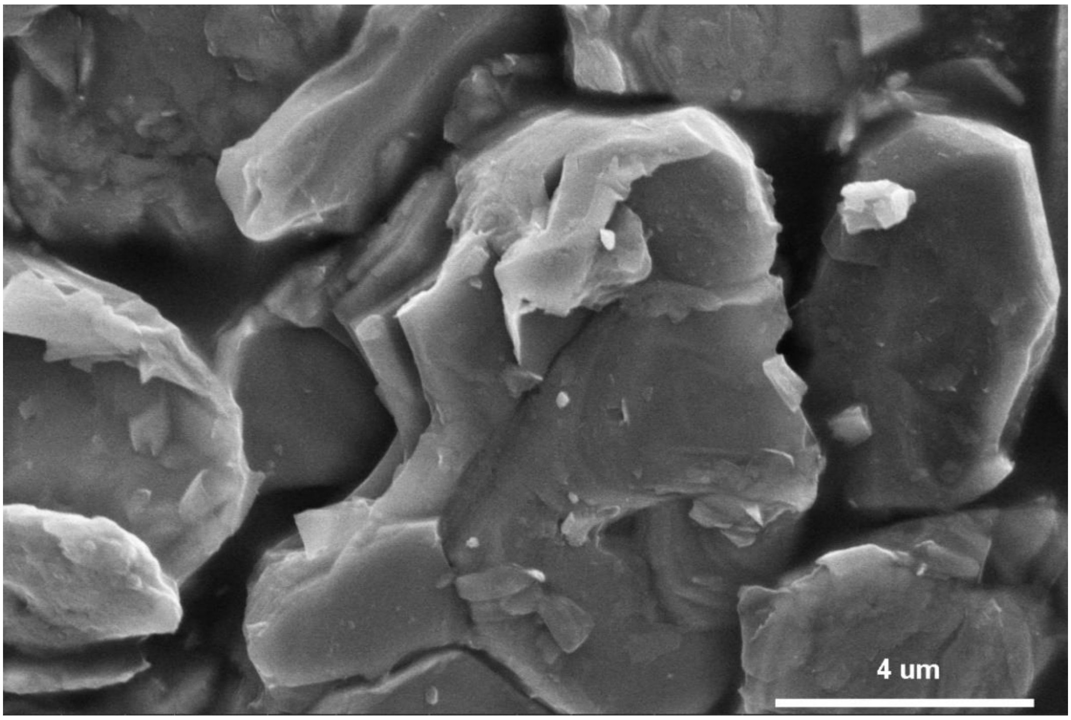

Figure S1. SEM image of Ti_3AlC_2 MAX phase.

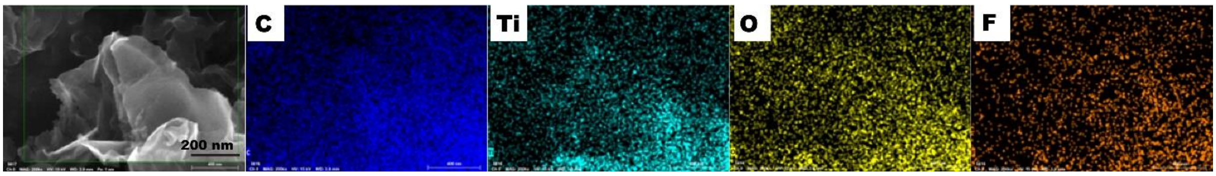

Figure S2. The morphology and EDS-maping of the pure sheet MXene (Ti_3C_2).

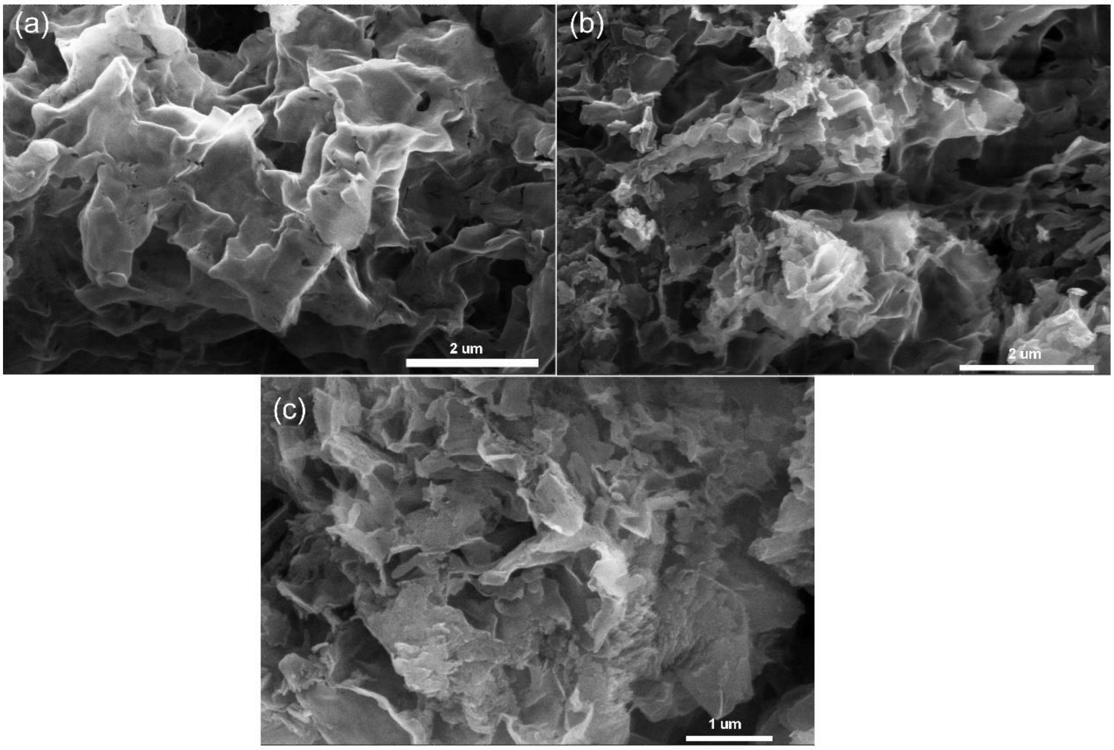

Figure S3. SEM images of 1-TC/CN, 3-TC/CN, 5-TC/CN.

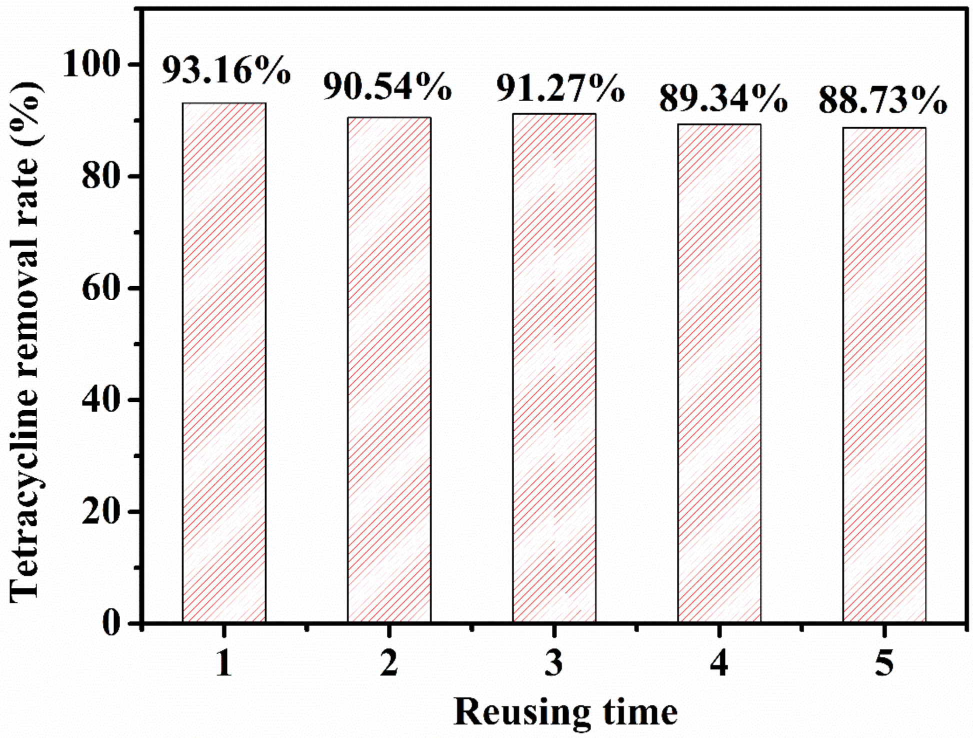

Figure S4. The the reusability of 2-TC/CN MXene composite.

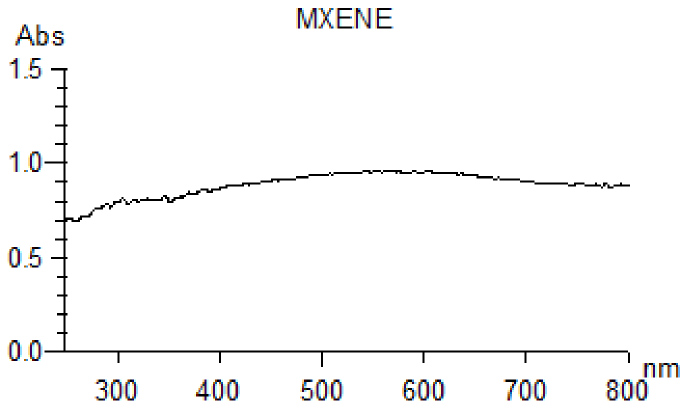

Figure S5. UV-vis spectra of the pure Mxene (Ti_3C_2).

Table S1. Valence band, band gap and conduction band computation procedure of the samples.

**Sample**	**λ (nm)**	**E_v (eV)**	**E_g (eV)**	**E_c (eV)**
$g\text{-}C_3N_4$	472.69	2.03	2.62	-0.59
1-TC/CN	538.57	1.91	2.30	-0.39
2-TC/CN	532.59	1.71	2.33	-0.62
3-TC/CN	509.94	1.82	2.43	-0.61
5-TC/CN	551.12	1.74	2.25	-0.51

Table S2. Comparison of the photocatalytic activity of degrading tetracycline of the prepared 2-TC/CN with that of photocatalysts in literature.

	**C_0(TC) [× 10^{-5} mol/L]**	**Dosage of Catalyst[g/ L]**	**Light source**	**degradation rate**	**Ref.**
**MCN-2**	2.5	0.3	350 W XL λ > 420 nm	75% (30 min)	[1]
**Ag/$g\text{-}C_3N_4$**	4.2	1.7	300 W XL λ > 420 nm	0.012	[2]
**Porous $g\text{-}C_3N_4$**	4.5	0.3	300 W XL λ > 420 nm	91.8% (60 min)	[3]
**2-TC/CN**	4.5	0.5	300 W XL λ > 420 nm	93.93% (60 min)	Our work

The PL spectra of the samples could be well fitted to the two-exponential model as the following expression. The fitting parameters are summarized in Table S2.

$$(A_1*\tau_1^2)+(A_2*\tau_2^2)/(A_1*\tau_1+A_2*\tau_2) \quad \text{(S1)}$$

Table S3. The calculation procedure of the lifetime of photogenerated carriers.

Parameter	$g-C_3N_4$	1-TC/CN	2-TC/CN	3-TC/CN	5-TC/CN
τ_1 (ns)	2.56145	1.75674	2.11815	2.36801	9.14025
A_1 (%)	52.23082	71.99474	77.87007	81.79226	9.5068
τ_2 (ns)	2.56155	6.00792	8.53001	11.09456	2.12202
A_2 (%)	52.23082	21.85589	10.53793	7.55754	85.39593
$(A_1*\tau_1^2)+(A_2*\tau_2^2)/(A_1*\tau_1+A_2*\tau_2)$	2.56 ns	3.92 ns	4.38 ns	5.00 ns	4.40 ns

References

1. Wang, Z.; Zhang, Y.; Yu, Y.; Jia M.; Tao, X. Promoting photocatalytic degradation of tetracycline over in-situ grown single manganese atoms on polymeric carbon nitride. *Appl. Surf. Sci.* **2022**, *593*, 153458.
2. Xu, W.; Lai, X.; Pillai, S.C.; Chu, W.; Hu, Y.; Jiang, X.; Fu, M.; Wu, X.; Li, F.; Wang, H. Visible light photocatalytic degradation of tetracycline with porous Ag/graphite carbon nitride plasmonic composite: degradation pathways and mechanism**.** *J. Colloid Interface Sci.* **2020**, *574*, 110–121.
3. Zhang, J.; Ma, Z. Porous $g-C_3N_4$ with Enhanced Adsorption and Visible-Light Photocatalytic Performance for Removing Aqueous Dyes and Tetracycline Hydrochloride. *Chin. J. Chem. Eng.* **2018**, *26*, 753–760.
